# Interacting Adaptive Processes with Different Timescales Underlie Short-Term Motor Learning

**DOI:** 10.1371/journal.pbio.0040179

**Published:** 2006-05-23

**Authors:** Maurice A Smith, Ali Ghazizadeh, Reza Shadmehr

**Affiliations:** **1**Division of Engineering and Applied Sciences, Harvard University, Cambridge, Massachusetts, United States of America; **2**Department of Bioengineering, University of California Berkeley, Berkeley, California, United States of America; **3**Department of Biomedical Engineering, Johns Hopkins University School of Medicine, Baltimore, Maryland, United States of America; University of MinnesotaUnited States of America

## Abstract

Multiple processes may contribute to motor skill acquisition, but it is thought that many of these processes require sleep or the passage of long periods of time ranging from several hours to many days or weeks. Here we demonstrate that within a timescale of minutes, two distinct fast-acting processes drive motor adaptation. One process responds weakly to error but retains information well, whereas the other responds strongly but has poor retention. This two-state learning system makes the surprising prediction of spontaneous recovery (or adaptation rebound) if error feedback is clamped at zero following an adaptation-extinction training episode. We used a novel paradigm to experimentally confirm this prediction in human motor learning of reaching, and we show that the interaction between the learning processes in this simple two-state system provides a unifying explanation for several different, apparently unrelated, phenomena in motor adaptation including savings, anterograde interference, spontaneous recovery, and rapid unlearning. Our results suggest that motor adaptation depends on at least two distinct neural systems that have different sensitivity to error and retain information at different rates.

## Abstract 2

This study presents evidence for two learning processes with distinct time courses that contribute to motor skill acquisition, and a computational model of the interactions between these processes that unifies much of the literature in motor adaptation.

## Introduction

Savings is a fundamental property of memory. It refers to the ability of prior learning to speed subsequent relearning even after behavioral manifestations of the prior learning have been washed out. A typical experiment that demonstrates savings has three parts. First, a novel response to a stimulus is gradually learned over the course of many trials. Next, this stimulus-response relationship is unlearned or extinguished so that the stimulus no longer evokes the learned response. Finally, the initially learned stimulus-response relationship is relearned under the original learning conditions. If savings is present, relearning will proceed more quickly than initial learning.

Savings has been studied in several classical conditioning [ 1] and operant conditioning paradigms [ 2] but until recently had not been demonstrated in motor adaptation. Motor adaptation is a type of learning in which motor commands are altered to compensate for disturbances in the external environment or in the motor system itself. A recent study of eye saccade gain adaptation by Kojima et al. [ 3] elucidated several properties of savings in motor adaptation. This study showed that (1) savings can occur in a motor adaptation task, (2) it can cause a sudden jump in performance if a block of no-feedback (dark) trials is inserted between the extinction and re-adaptation blocks, and (3) it can be washed out if a block of baseline trials is inserted between the extinction and re-adaptation blocks.

Current models of trial-to-trial motor adaptation cannot account for these results. While these models have been successfully used to predict motor responses to novel, randomly generated disturbance sequences [ 4, 5] and to assess the patterns of generalization [ 6, 7], these models all predict a single time constant of adaptation. However, in addition to savings, several other experimental observations suggest that time constants of adaptation may increase or decrease from baseline depending on the specifics of the training regimen. One well-known effect is anterograde interference. This refers to the finding that learning an initial motor adaptation reduces not only the initial performance but also the time constant for subsequently learning the opposite adaptation [ 8– 10]. Two other important observations are rapid de-adaptation [ 10, 11] and rapid downscaling [ 10], where fully or partially unlearning a motor adaptation can be faster than initial learning of this adaptation. In summary, current models of trial-to-trial adaptation fail to account for the effects of savings, spontaneous recovery, anterograde interference, rapid unlearning, and rapid downscaling.

To account for the results of their savings experiments, Kojima et al. suggested a novel two-state model in which distinct mechanisms specialized in increasing the gain of saccades versus decreasing it [ 3]. This gain-specific model successfully produced savings and washout of savings observed by these authors. However, it failed to account for the spontaneous recovery of the initially adapted state when monkeys were held in darkness following the extinction trials (allowing saccades to take place without error feedback). This model is also unable to explain the phenomenon of anterograde interference in which secondary learning is slower than baseline.

Saccade adaptation in monkeys [ 12– 14] and humans [ 15, 16], as well as a host of motor adaptation paradigms [ 17– 20], depends on the cerebellum. In the cerebellum, motor state and error information simultaneously arrive in two structures: the cerebellar cortex and the cerebellar nuclei. We wondered whether a two-state model in which each state learned at a different rate, rather than in a different direction, might be able to account for the full pattern of savings these authors saw, and simultaneously explain the effects of anterograde interference, rapid unlearning, and rapid downscaling. Here we show this to be the case.

## Results

**Figure 1:**
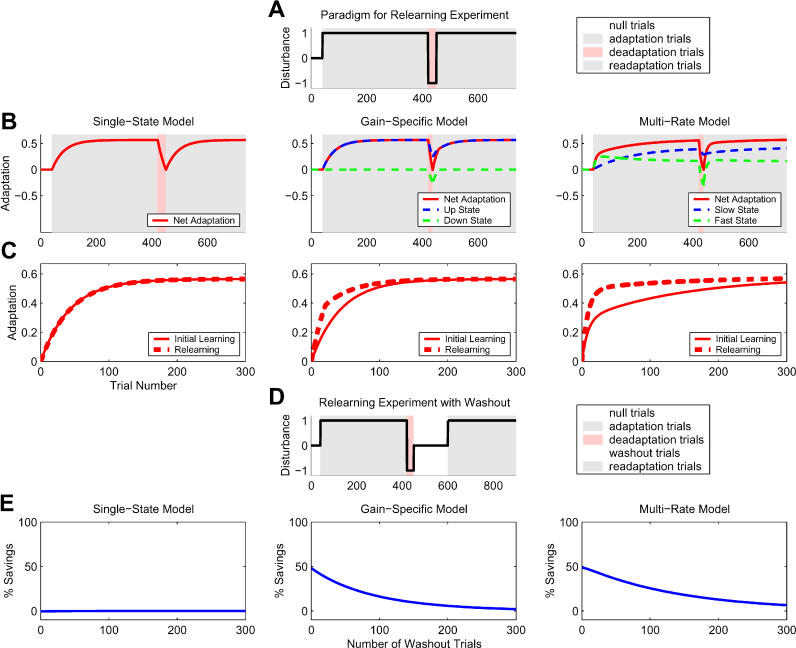
Simulations of Motor Adaptation Experiments That Show Savings (A) Paradigm for basic savings experiment. This paradigm consists of four blocks: (1) a baseline period, (2) initial learning, (3) unlearning, and (4) relearning. Note that adaptation stimulus for the unlearning block is opposite that used in learning blocks, and the number of trials in the unlearning block is adjusted so that on this block's last trial performance is at the baseline level. (B and C) Model simulations of the experiment paradigm shown in (A). The first row (B) shows the raw results of these simulations, while the second row (C) shows a direct comparison of simulated performance in the initial learning and relearning blocks. The different columns display simulation results from the single-state, gain-specific, and multi-rate models, respectively. The single-state model fails to show savings (faster relearning), but the gain-specific and multi-rate models show savings. (D) Paradigm for savings experiment with washout. Note that this paradigm is similar to the paradigm shown in (A), except that a washout block of variable length is inserted prior to the relearning block. (E) The amount of savings found in simulation, as a function of the number of washout trials. The amount of savings is measured as the percent improvement in performance on the 30th trial in the relearning block compared to the 30th trial in the initial learning block. The columns are the same as in (B). The gain-specific and multi-rate models show similar washout of savings; however, in the single-state model there is no savings to wash out.



Figure 1 shows simulations of the experimental paradigm that demonstrated savings in saccade adaptation [ 3]. We simulated the progression of motor output in this learn-unlearn-relearn paradigm with three different models: (1) a single-state, single time-constant model, (2) a two-state, gain-specific model, and (3) a two-state, gain-independent, multi-rate model. All of these models modify motor output on a particular trial as a function of the current motor state and the error experienced on that trial (see Materials and Methods). The learning rules for these models are shown below:

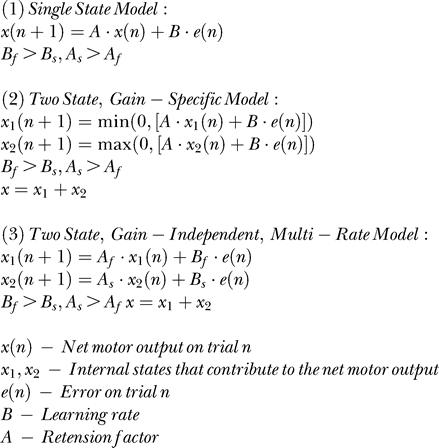



In all these models error arises because there is a difference between the motor output *x*( *n*) and the state of the environment *f*( *n*) such that: *e*( *n*) = *f* ( *n*) − *x*( *n*). While a single-state system cannot reproduce a motor output pattern that shows savings, both the gain-specific model proposed by Kojima et al. and our multi-rate model produce savings ( Figure 1A and 1B). Furthermore, both models predict decay in the amount of savings if null trials are inserted before the learning block ( Figure 1C and 1D). The key to faster relearning in both two-state models is that although net motor output is near zero at the beginning of the relearning block, the internal states are both non-zero. Because the internal states are different, both systems' responses to the learning stimulus are altered. Relearning is faster than initial learning in the gain-specific model because both the up and down states can contribute to relearning whereas only the up state contributes to initial learning. In the case of the multi-rate model, relearning is faster than initial learning because when relearning starts, the slow state is already biased towards relearning, making relearning more dependent on the fast state compared to initial learning.

**Figure 2:**
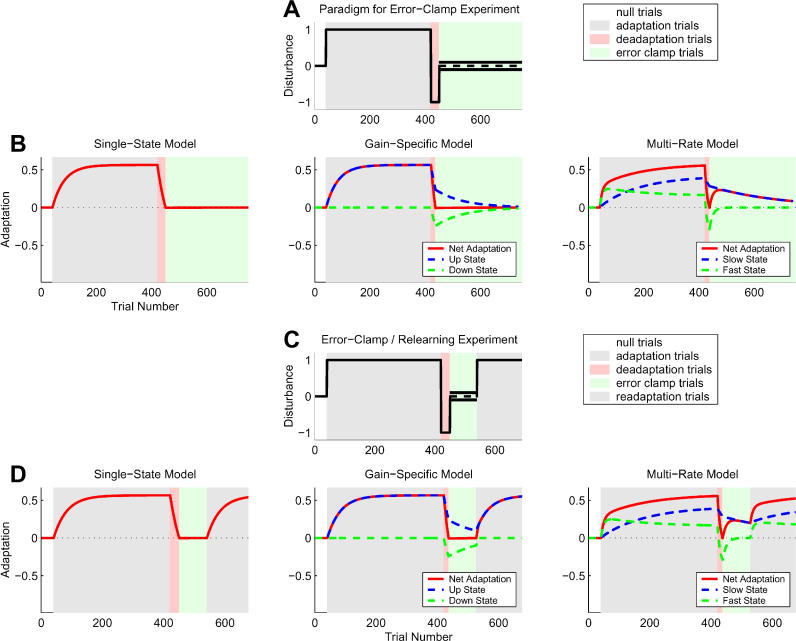
Simulations of Motor Adaptation in the Error-Clamp Paradigm (A) Paradigm for simulated error-clamp experiment. This paradigm is similar to the savings paradigm shown in Figure 1A except that the relearning block is replaced by an error-clamp block during which the error that drives adaptation is held at zero. (B) Model simulations of the experiment paradigm shown in (A). The different columns display simulation results form the single-state, gain-specific, and multi-rate models, respectively. The single-state and gain-specific models do not predict changes in motor output from baseline in the error-clamp block, whereas the multi-rate model predicts a transient rebound of motor output in the error-clamp block. This rebound is in the direction of the motor output displayed in the initial learning block, resulting in spontaneous recovery. (C) Paradigm for the error-clamp/relearning experiment. Here a relearning block follows a shortened error-clamp block. This paradigm reproduces the effect of jump-up facilitation seen by Kojima et al. following dark exposure. During dark exposure, monkeys made saccades but received no visual feedback of saccade error. The absence of error feedback may be similar to the zero-error condition produced by the error-clamp. (D) Model simulations of the experiment paradigm shown in (C). The columns are the same as in (B). The multi-rate model predicts that performance at the start of the relearning block is already better than baseline. This jump-up in performance is caused by adaptation rebound in the error-clamp phase. Kojima et al. showed that following a period of dark exposure (during which saccade gain was not measured) monkeys displayed an immediate jump-up in performance at the start of the subsequent relearning block. This finding is predicted by the multi-rate model, but is not predicted by the single-state or gain-specific models.

Although the gain-specific and multi-rate models predict similar patterns of behavior following extinction, their internal states evolve quite differently. This suggests that these models may make different predictions about the pattern of motor output on other experimental paradigms. We found that if the relearning phase of the learn-unlearn-relearn experiment is replaced by a zero-error block, i.e., if the error is clamped at zero following the unlearning block, the gain-specific and multi-rate models can make very different predictions about the evolution of motor output. These predictions are shown in Figure 2. The gain-specific model predicts that following the unlearning block, motor output will remain at zero if error is clamped at zero. In contrast, the multi-rate model predicts a rebound effect, or spontaneous recovery, during this same period. Instead of remaining at zero, predicted motor output during the zero-error block transiently rebounds toward the motor output during the initial learning block ( Figure 2B). This produces an apparent jump in performance when performance is measured at the end of the error clamp period ( Figure 2D)—something that Kojima et al. observed in their saccade adaptation experiment following a period of darkness [ 3]. Interestingly, phenomena similar to the spontaneous recovery produced by the multi-rate model have been observed in classical conditioning experiments following extinction training in animals [ 21– 23].

Inspection of the internal state dynamics of the multi-rate model reveals that this phenomenon occurs because the fast learning module rapidly decays to zero during the error-clamp block, while the slow learning module decays more gradually. The transient rebound occurs as the fast learning module decays while the slow learning module is mostly intact, and this rebound fades as the slow learning module progressively decays. In a saccade adaptation paradigm where lights are turned off, it is not possible to observe the errors that the animal makes and therefore these transients cannot be measured. However, we designed a different adaptation paradigm that allowed us to clamp motor error at zero and yet directly measure the transients of the motor output and therefore test the predictions of our model.

We implemented the error-clamp paradigm in a force-field adaptation experiment where individuals reached to a target (see Materials and Methods). Human participants adapted to a viscous-curl force field imposed on their hands by a robot manipulandum. Like saccade adaptation, force-field adaptation is also thought to depend on the cerebellum [ 19, 20] and is known to produce quick, robust, easily measured motor learning [ 24]. We used a robotic arm to apply clockwise and counterclockwise viscous-curl force fields [ 25] to induce adaptation and de-adaptation in point-to-point goal-directed voluntary reaching movements (see Materials and Methods). In this experiment we also used the robot arm to create a virtual force channel in order to clamp lateral errors to zero on selected trials [ 26]. In these trials, perpendicular displacement from a straight-line movement was held to less than 0.6 mm, limiting the size of perpendicular errors to very small values compared to when the force channel was not applied. We trained 14 individuals in one force field (where forces pushed their hands perpendicular to the direction of motion) and then switched them to the opposite force field for a short period. We then switched them again to error-clamp trials to record the changes in lateral forces that these individuals produced when lateral error was held near zero. As in the simulations shown in Figure 2, the second field acted as a washout on the first field so that reaching movements in the channel started with near zero lateral force.

**Figure 3:**
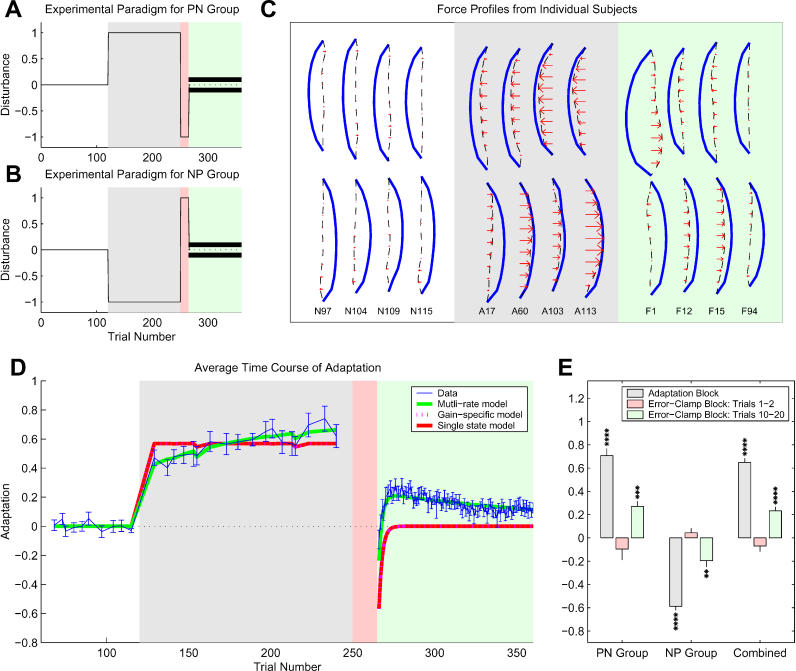
Experimental Results from the Force-Field Learning Error-Clamp Paradigm (A and B) Paradigms for simulated error-clamp experiment. These paradigms are the same as the paradigm shown in Figure 2A, except that here one group (the NP group) of participants is exposed to an initial adaptation to a clockwise viscous-curl force field while the other group (the PN group) is exposed to an initial adaptation in the opposite direction (counterclockwise). (C) Example force trajectories during the course of this learning paradigm. Force trajectories from selected error-clamp trials for one participant in each group are shown as red arrows with tips connected by dashed black lines. The blue line represents the force trajectory required to fully cancel the force field applied during the initial learning block for each participant. The same trials are shown for each participant, and each trial is labeled by a block identifier and the trial number within that block. For example, N97 is the 97th trial in the null-field practice block, A17 is the 17th trial in the initial adaptation block, and F1 is the first trial in the force-channel (error-clamp) block. Since the adaptation requires the production of lateral forces, only lateral forces are shown. Lateral forces (red arrows) in the baseline period are small and inconsistent in direction. However, during the initial adaptation block these lateral forces grow with training so that they nearly cancel the applied force field. After the extinction block, the first trials in the error-clamp block show a near-zero or negative pattern of lateral forces with respect to the forces displayed late in the initial adaptation block. However, by trials 12–15 in the error-clamp block, a small but consistent rebound of the pattern of lateral forces seen during initial adaptation emerges. This rebound substantially fades away by trial 90 in the error-clamp block. (D) The average time course of adaptive changes in the pattern of lateral forces. Data from both the PN and NP groups are averaged together. The adaptation score corresponding to the force pattern displayed on a particular trial was assessed by computing a force-field compensation factor (see Materials and Methods). In short, this force-field compensation factor measures the fraction of (initial adaptation) force field that would be compensated by the pattern of lateral forces displayed on a particular trial by regressing the measured lateral force pattern onto the ideal pattern of lateral forces required to fully compensate the force field. The transient rebound of motor output in the error-clamp block matches the rebound predicted by the multi-rate model. The blue error bars represent experimental data (mean +/− standard error of the mean.). The green line is the best-fit multi-rate model, and the red and purple lines are the best-fit gain-specific and single-state models. The best-fit model parameters (with 95% confidence intervals) for the multi-rate model were A _1_ = 0.992 (0.990–0.994), B _1_ = 0.02 (0.013–0.025), A _2_ = 0.59 (0.43–0.76), and B _2_ = 0.21 (0.10–0.35). (E) Summary of results from NP and PN groups. The asterisks indicate significant difference in lateral forces from baseline. Both groups display significant adaptation rebound by trials 10–20 of the error-clamp block compared to the initial error-clamp trials ( *p* < 0.01 for both the NP and PN groups taken separately, and *p* < 0.0001 for all participants taken together) and compared to baseline lateral force levels before learning ( *p* < 0.01 for the NP group, *p* < 0.001 for the PN group, and *p* < 0.0001 for all participants taken together). NP, negative/positive group; PN, positive/negative group.

Individual participant data displayed in Figure 3 shows that participants learned the initial force field well. Late in training participants produced a pattern of lateral forces that closely matched the ideal force pattern required to fully compensate the applied force field. Although this pattern of forces was unlearned in the second force-field block so that the first error-clamp trial showed small or oppositely directed forces, as predicted by the multi-rate model, the initially learned force pattern reemerged by trials 12 and 15.

We quantified the extent of force-field adaptation during force channel trials by regressing the force profile produced orthogonal to the force channel onto the force profile required to make the same straight-line movement in a viscous-curl force field (see Materials and Methods). This regression yielded a factor which estimated the fraction of the force field compensated in each error-clamp trial. The force-field compensation was zero or negative during the first few trials in the error-clamp block indicating complete unlearning during the second force-field block. However, force-field compensation rapidly rebounded toward the initial adaptation in the error-clamp block independent of the direction of initial learning ( *p* < 0.0001 for all 14 participants taken together, *p* < 0.01 for each seven-participant subgroup taken separately, both when compared to the initial error-clamp trials and when compared to zero lateral force (see Figure 3E). The rebound grew during the first ten to 20 error-clamp trials and then gradually declined toward zero. This rebound effect was predicted by the multi-rate model but not the gain-specific model or the single-state model as shown in Figure 3D. Furthermore, the time course of the rebound—a rapid rise then a slow decline—was also predicted by the multi-rate model.

These findings suggest that within minutes of training on the adaptation task, multiple learning modules contribute substantially to the process of force-field adaptation. In fact, the slow learning module accounts for more than half of the total adaptation by the end of the first learning block, suggesting that this module may play a significant role in even short-term motor adaptation tasks.

It should be noted that if the gain-specific model is expanded to allow asymmetric learning rates and forgetting factors (see Supporting Information ), it can in some cases produce rebound. However, this rebound is quite different from the spontaneous recovery produced by the multi-rate model, because this rebound will always be in the direction of the gain process with the slower forgetting factor rather than in the direction to recover previous learning, as predicted by the multi-rate model and displayed in our data (see Figure 3E).

**Figure 4:**
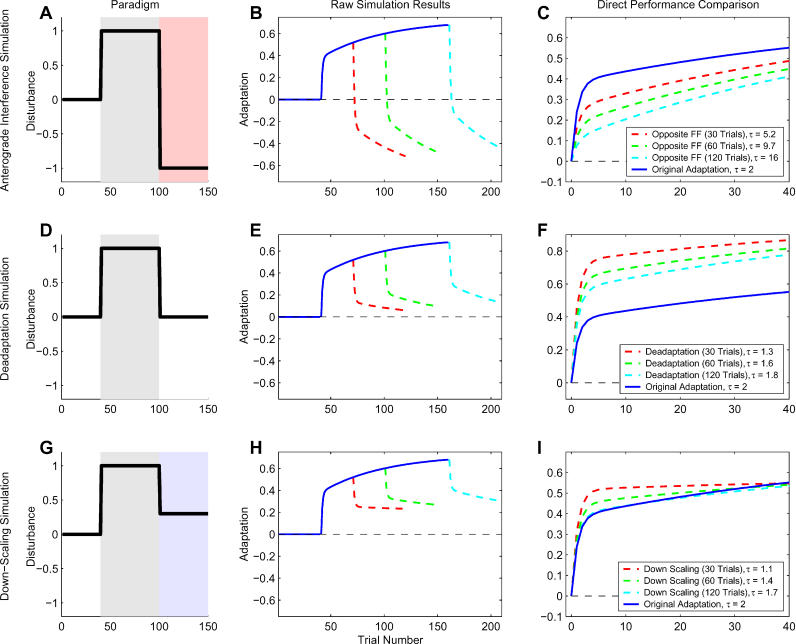
Simulations of Motor Adaptation with the Multi-Rate Model Explain a Variety of Previously Reported Results, Including Rapid Unlearning and Rapid Downscaling (A–C) Anterograde interference. (D–F) Rapid unlearning. (G–I) Rapid downscaling. First column (A, D, and G): experiment paradigms. Second column (B, E, and H): Raw simulation results. Blue: initial adaptation. Red, green, and cyan: secondary adaptation after 30, 60, or 120 trials of the initial adaptation, respectively. Third column (C, F, and I): Comparison of adaptation rates for initial and secondary adaptations. Here the learning curves have been shifted so that they all begin at zero and scaled so that the desired performance level is one. In the anterograde interference paradigm (A–C), the multi-rate model predicts that learning the opposite force field proceeds with a slower time constant than initial learning; furthermore, this time constant gets even slower when number of trials in the initial learning block is increased. The multi-rate model predicts that unlearning proceeds with a faster time constant than initial learning (E–F) and the time constant for downscaling is faster still (H–I); however, the time constant for unlearning or downscaling returns to baseline when the number of trials in the initial learning block is increased. In summary, the multi-rate model simultaneously explains the effects of anterograde interference, rapid unlearning, and rapid downscaling. Furthermore this model predicts that anterograde interference will get stronger as the length of the initial adaptation period increases, but that rapid unlearning and rapid downscaling will get weaker as the length of the initial adaptation period increases.

The multi-rate model accounts for several other well-known phenomena that have been observed in motor adaptation ( Figure 4). For example, studies have shown that the time constant for an initial motor adaptation is faster than the time constant for subsequent adaptation to the oppositely directed adaptation stimulus [ 8– 10]. This effect has been termed anterograde interference ( Figure 4A–C). The single-state model and the gain-specific model are unable to explain anterograde interference (see Supporting Information for simulation results). The single-state model predicts no effect of anterograde interference, while the gain-specific model actually predicts that the time constant for the second adaptation will be faster than that of the first. However, the multi-rate model correctly predicts slower learning of the second adaptation in the interference paradigm. The multi-rate model predicts slower learning because the slow learning module is initially biased against learning the second adaptation.

Several studies have shown that when a motor adaptation is learned then washed out, the rate of de-adaptation back to baseline can be faster than the rate of initial adaptation [ 10, 11]. The multi-rate model not only explains this effect (which is also predicted by the gain-specific model), but explains why the apparent magnitude of this effect can vary substantially from one paradigm to another ( Figure 4D–F). Our model predicts that the amount of facilitation in the time constant for de-adaptation will be maximal after fairly short adaptation blocks and then decline as the duration of adaptation increases.

Finally, Davidson and Wolpert recently reported that the time constant for adapting to a scaled down version of a previously learned force-field adaptation can be even faster than the rate of de-adaptation to baseline [ 10]. This effect can also be explained by the multi-rate model ( Figure 4G–I) but not by the single-state or gain-specific models.

**Figure 5:**
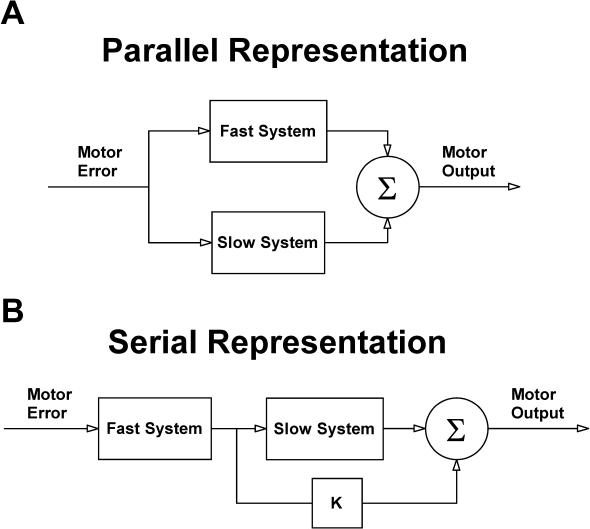
Two Different Internal Realizations of a Linear, Two-State, Multi-Rate System Any input-output behavior achieved by one realization can be duplicated by the other.

Our multi-rate model is a member of the general class of multi-state single-input, single-output linear state-space models. One important feature of this class is that multiple realizations of the same input-output behavior are possible, i.e., internal system architectures are not unique. Of particular interest are the two equivalent system architectures diagrammed in Figure 5. In the first representation, two learning modules independently adapt from error and their outputs are combined to produce changes in net motor output. In the second representation, the two learning modules are cascaded such that the fast module adapts directly from error while the slow module adapts indirectly via the output of the fast module. Because these representations can have identical input-output behavior, behavioral experiments alone in animals or people with normally functioning motor learning systems cannot distinguish them. However, the combination of behavioral experiments with neurophysiology and lesion studies may be able to extract the neural architecture of this multi-rate system.

It should be noted that the models presented here are written in terms of trial number with no explicit effect of time. However, the decay terms in our model could account for both trial-related decay and the average time-related inter-trial decay. Because these models are written purely as functions of trial number, they imply that the trial-to-trial decay in motor memory is primarily related to the passage of trials per se rather than to the passage of time during the inter-trial intervals. We write the models in this way because the studies that have looked at motor memory retention in short-term motor adaptation have showed little change in motor memory with the passage of time alone for periods up to an hour, but significant memory decay when trials without error feedback were applied [ 27, 28]. Although this evidence suggests that the trial-related decay is dominant, since we did not explicitly test the effect of time on memory decay in our models, we cannot rule out that it has some effect.

## Discussion

Here we have presented evidence that short-term motor adaptation is substantially influenced by two adaptive processes with different learning rates and different capacities for retention. It is clear that at least part of this multi-rate system is dependent on or contained within the cerebellum. Patients with cerebellar lesions from a variety of causes [ 15, 17– 20], as well as animals given cerebellar lesions [ 29], show dramatic deficits in the rate of motor adaptation, but it is unclear whether motor adaptation in these patients is entirely absent or occurs at a markedly reduced rate matching that of the slow module in our model. This suggests that at least the fast learning module—if not both—is strongly dependent on the cerebellum for normal function.

Medina et al. have shown that a coarse response to classical conditioning of the eye-blink reflex develops in the cerebellar interpositus nucleus in rabbits gradually over days of training, although the overall time course of the learning is much faster [ 1]. Furthermore, the magnitude of this slowly developing response correlates with the amount of savings (improved relearning) after the conditioned response has been extinguished. Although the development of this response occurs much more slowly than the slow component of the response in our present data, the lag behind performance improvement and the relationship to savings suggest that during eye-blink conditioning in rabbits, the cerebellar nuclei may act very much like the slow learning module in our model of motor adaptation; albeit with an even more gradual response, while the cerebellar cortex acts like the fast learning module. Interestingly, current lesion experiments in the eye-blink reflex support the cascade model of adaptation where error rapidly teaches the cerebellar cortex while the cerebellar cortex slowly teaches the nucleus [ 30].

The learning modules from our model may also depend on motor areas other than the cerebellum. For example, neural recordings from motor cortex during a force-field adaptation task in highly trained monkeys show distinct “memory cells” despite evidence of behavioral extinction [ 31]. These neural responses during extinction show that the cells fall into two classes such that the sum of the contributions of the two classes adds to zero, while each class predicts a different pattern of force. The multi-rate model predicts that the “memory I” cells reported by these authors are a reflection of the slow system; showing strong adaptive responses by the end of initial training that are maintained during extinction. In contrast, our model predicts that the “memory II” cells are a reflection of the fast system, showing little or no adaptive response by end of initial training but a strong response during extinction (in order to compensate for the slow system). Therefore, whether the modules that we see depend on the cerebellum, motor areas in the cerebral cortex, both, or even other cortical or subcortical structures, is at this point unclear.

In fact, the fast and slow adaptive processes that we have inferred from the data do not necessarily implicate separate neural systems, but might even be part of the adaptive mechanisms of single synapses or single neurons. For example, the probability of change in a synapse may strongly depend on its prior history of stimulation, as modeled recently by Fusi et al. [ 32] (see simulations of this model in Supporting Information). Alternatively, a step change in a stimulus' properties may produce changes in firing rates of single cells that are not step-like or single exponential, but show adaptation with multiple timescales [ 33].

It is likely that the neural apparatus for motor adaptation has functional modules with even more than two different time courses. Here we examined short-term, single-session motor adaptation, found evidence for two distinct time courses, and showed that the properties of a simple linear system with two time courses provides a single, unified explanation for a wide variety of phenomena in short-term motor adaptation. The phenomena include savings, anterograde interference, spontaneous recovery, rapid unlearning, and rapid downscaling. However, studies of memory consolidation during motor learning suggest that additional processes with slower, even more gradual time courses may play important roles during long-term motor learning. Understanding the interplay between these different processes will give us fundamental insights into understanding motor memory formation.

## Materials and Methods

### Modeling.

We used the learning rules for the single-state, gain-specific, and multi-rate models shown in the main text along with the error equations below to iteratively compute the time course of adaptation for each model in each simulated experiment.



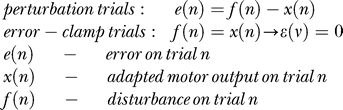



For the simulations shown in Figures 1 and 2, the model parameters were arbitrarily set at A = 0.99 and B = 0.013 for the single-state and gain-specific models; and A _f_ = 0.92, A _s_ = 0.996, B _f_ = 0.03, and B _s_ = 0.004 for the multi-rate model. However, the qualitative results that we describe here do not depend on these particular parameter values; they hold as long as all parameters are positive, and B _f_ is several-fold larger than B _s_, and A _s_ is several times closer to one than A _f_ (see Supporting Information for an analysis of the effect parameter variation for the gain-specific model). Each of the plots showing washout of savings in Figure 1E display data derived from a series of 301 simulations. The number of washout trials was varied from 0 to 300, and the percent savings was computed for each simulation as the performance improvement on trial 30 of the relearning block versus trial 30 of the initial learning block.

In Figure 3D we find the parameter values for each model that best fits the data in a least-squares sense, and we use these parameter values for the multi-rate model to make the model predictions shown in Figure 4. To compute the time constants displayed in Figure 4 we fit the first 50 trials of the simulation results in the primary and secondary adaptation blocks with a single exponential function and extracted its time constant. We computed confidence intervals on the best-fit parameter values by bootstrapping model fits to the data. We made 1,000 different bootstrap estimates of the data mean, each by averaging data from 14 randomly generated choices made from the 14-participant data pool with replacement. We fit the model to each of these bootstrap estimates and used the 2.5 and 97.5 percentile values of each parameter as the limits of the 95% confidence interval.

### Participants.

14 healthy participants (mean age 24) without known neurological impairment were recruited from the Johns Hopkins Medical School community. All participants were right handed and used their dominant hands. All participants gave informed consent and the experimental protocols were approved by the Johns Hopkins Institutional Review Board.

### Task.

We studied a variant of the standard force-field adaptation paradigm [ 24]. Briefly, participants held the handle of a two-joint manipulandum that could move in the horizontal plane. A small round cursor (3 mm in diameter) indicated the participant's hand position and was displayed on a vertically oriented computer monitor in front of the participant (refresh rate of 75 Hz). They reached to circular targets 1 cm in diameter that were spaced 10 cm apart. The manipulandum measured hand position, velocity, and force, and its motors were used to apply forces to the hand, all at a sampling rate of 200 Hz.

Four trial types were used: null trials, force-channel trials, clockwise curl-field trials, and counterclockwise curl-field trials. Null trials were used for initial practice. During these trials the robot motors were turned off. During force field trials, the motors were used to produce forces on the hand that were proportional in magnitude and perpendicular in direction to the velocity of hand motion. The relationship between force (F) and velocity (V) vectors was determined by the matrix *C _A_ =[0 13;-13 0] Ns/m* via the relationship F = *C _A_* × V. We considered two kinds of fields: a clockwise curl-field *C _A_* and a counterclockwise curl- field *C _B =_ −C _A_*. We refer to these force fields as field A and field B, respectively. During force channel trials, the robot motors were used to constrain movements in a straight line toward the target by effectively counteracting any motion perpendicular to the target direction. This was achieved by applying a stiff one-dimensional spring (6 kN/m) and damper (150 Ns/m) in the axis perpendicular to the target direction. This error clamp was quite effective. In these trials, perpendicular displacement from a straight line to the target was held to less than 0.6 mm and averaged about 0.2 mm in magnitude.

The experiment was divided into short sets of 120 trials, each a reach to a target (60 reaches in each direction). Sets generally took 5–7 min to complete. There were two possible target locations 10 cm apart in the body midline such that odd-numbered trials were directed toward the body and even numbered trials were away from it. The force channel was applied on all outward reach trials for the entire experiment. The inward reach trials were performed under several different conditions as follows: The first two sets were performed in the null field with the robot motors disabled. The next two sets were performed in the first force field. The fifth set consisted of ten trials in the first force field, followed by 15 trials in the opposite force field, and then 35 consecutive force channel trials. The sixth and final set consisted of 60 consecutive force channel trials. In sets 2–4, nine force-channel trials (about one in seven) were randomly interspersed among the null or force field trials to measure the progression of force-field adaptation. The 14 participants were randomly assigned into two counter-balanced groups of seven, such that one group, the negative/positive (NP) group, first experienced the clockwise force field and then experienced the counterclockwise force field, whereas the positive/negative (PN) group, first experienced the counterclockwise force field and then experienced the clockwise force field.

We instructed participants to “make quick movements to the targets.” We instructed them that the reaction time was not important—they could wait as long as they wished after target appearance before starting each movement—but when ready, they were to move in a rapid motion toward each target. The endpoint of each movement was used as the starting point for the subsequent movement, and movements were made in two target directions.

### Analysis of force profiles.

Since the environmental perturbations applied during this experiment consisted of forces perpendicular to the direction of motion, we focused our analysis on the lateral force profiles that participants generated during movement. In general, lateral force could reflect an adaptive compensation of expected lateral force or an online corrective response to errors detected during the course of movement. Specifically, we looked at the progression of lateral force profiles during error-clamp trials in the null, initial learning, and error-clamp blocks of the experiment. During these trials, lateral errors were kept small (less than 0.5 mm), so lateral force profiles essentially reflected adaptive compensation of the force-field perturbations. Since full compensation of the force-field perturbation on a particular trial required a lateral force profile proportional to the speed profile on that same trial (and this speed profile varied from one trial to another), we assessed the amount of adaptation on each error-clamp trial by computing a force-field compensation factor found by linear regression of the measured lateral force profile onto the ideal force profile required for full force-field compensation on that trial. This force-field compensation factor was zero if these force profiles were uncorrelated and one if these force profiles were identical to one another.

## Supporting Information

Combined Supporting Information (625 KB DOC)
