# Supplementary material for: Interacting Adaptive Processes with Different Timescales Underlie Short-Term Motor Learning
Source: PLoS Biol. 2006 May 23;4(6):e179. doi: 10.1371/journal.pbio.0040179 (PMC1463025; doi:10.1371/journal.pbio.0040179)
Supplement: Supplementary file 1 [file pbio.0040179.sd001.doc]

**Supplementary materials:**

**Content:**

Predictions of the multi-rate model compared to the predictions of the gain-specific and single-state models.

Relationship between an error-dependent and a force-dependent learning model.

Simulation of an asymmetric gain-specific model of learning.

Robustness of spontaneous rebound in the multi-rate model: simulation of the effects of different learning rates and forgetting factors on the amount spontaneous rebound predicted by the multi-rate model.

Simulation of a cascade memory model of synaptic plasticity.

**The relationship between force-dependent and error-dependent multi-rate learning models:**

In this paper we modeled motor adaptation as a phenomenon driven by the kinematic motor errors experienced. However, it is possible to think of motor learning as a process driven by external force (or more generally, by an external disturbance) rather than by the motor error that this disturbance causes. Here we show that there is a clear analytical relationship between these two formulations of the learning process. Therefore, our error feedback model can be perfectly recast into the framework of force feedback, so that learning in each trial depends on sensed or estimated external force rather than sensed error. This analysis shows that force-dependent learning is functionally equivalent to error-dependent learning with a simple change of coordinates standing between them. In general, for any stable error-dependent linear learning rule, a fully equivalent force-dependent learning rule exists that will produce an identical pattern of learning. Furthermore, the following analysis will show that the number of internal states (or, loosely, the complexity) in these two representations will be identical.

**Figure S1**: Relationship between error-dependent and force-dependent learning systems

Figure S1 shows that force-dependent learning is equivalent to error-dependent learning occurring in a closed loop. The loop is closed in error-dependent learning because the input to the learning modules (error) depends in part on the motor output. Thus force-dependent learning is the open-loop counterpart to error-dependent learning.

For the force-feedback version of the model, the fast and slow components of the system’s responses to a constant force disturbance, $f$, are both single exponentials in trial number. The fast force-feedback system produces a motor output pattern that is fast exponential with a time constant of $1/(1-A'_{f})$movements and an asymptote of $f\cdot[B'_{f}/(1-A'_{f})]$, while the slow force-feedback system produces a motor output pattern that is slower exponential with a time constant of $1/(1-A'_{s})$movements and an asymptote of $f\cdot[B'_{s}/(1-A'_{s})]$, where $B'_{f}, A'_{f}, B'_{s}, and A'_{s}$are the learning rates and retention factors for a force-dependent learning system as defined in equations s13-s15 below. Since the net motor output is the sum of these single-exponential responses, the relationship between our multi-rate model and a simple double exponential fit of the learned response to a constant force disturbance is clear: in a double exponential fit to data from a constant force disturbance, each exponential corresponds directly to the response of one component of the force-feedback representation of the learning system. Similarly, in a double exponential fit to data from a constant error disturbance (error clamp), each exponential corresponds directly to the response of one component of the error-feedback representation of the learning system.

Equations s1 through s4 show the trial-to-trial learning rules for the error-dependent multi-rate learning model, and the relationship between external force ($f$), motor output ($x$), and error($e$).

$$\begin{matrix} (s1) e(n)=f(n)-x(n) \\ (s2) x_{f}(n+1)=A_{f}x_{f}(n)+B_{f}e(n) \\ (s3) x_{s}(n+1)=A_{s}x_{s}(n)+B_{s}e(n) \\ (s4) x(n)=x_{f}(n)+x_{s}(n) \\ \\ (s5) G_{f}(z)=\frac{X_{f}(z)}{E(z)}=\frac{B_{f}}{z-A_{f}} \\ (s6) G_{s}(z)=\frac{X_{s}(z)}{E(z)}=\frac{B_{s}}{z-A_{s}} \\ (s7) G(z)=\frac{X(z)}{E(z)}=\frac{B_{f}}{z-A_{f}}+\frac{B_{s}}{z-A_{s}}=\frac{(B_{f}+B_{s})z-(A_{f}B_{s}+A_{s}B_{f})}{z^{2}-(A_{f}+A_{s})z+A_{f}A_{s}} \end{matrix}$$

The z-transform transfer functions $G_{f}(z)$, $G_{s}(z)$, and $G(z)$ displayed in equations s5 to s7 capture the relationship between error and motor output corresponding to the learning rules in equation s2 to s4. The relationship between the transfer function $G(z)$that relates motor output to error and the transfer function $H(z)$that relates motor output to external force is given in equation s8.

$$(s8) H(z)=\frac{X(z)}{F(z)}=\frac{G(z)}{\begin{matrix} 1+G(z) \\ \end{matrix}}=\frac{(B_{f}+B_{s})z-(A_{f}B_{s}+A_{s}B_{f})}{z^{2}-(A_{f}+A_{s}-B_{f}-B_{s})z+A_{f}A_{s}-A_{f}B_{s}-A_{s}B_{f}}=\frac{B'_{f}}{z-A'_{f}}+\frac{B'_{s}}{z-A'_{s}}$$

If$H(z)$is written in the final form shown in equation 8 (the fifth expression in equation 8), the constants $A'_{f}$, $A'_{s}$, $B'_{f}$, and $B'_{s}$ correspond to the learning rates and retention factors for force-dependent trial-to-trial learning rules shown in equations s13 to s15. The relationships between these new constants and the learning rates and retention factors for the error-dependent trial-to-trial learning rules are shown in equations s9 to s12. Using the z-transform these relationships can be derived by straight forward algebraic manipulation. Specifically, these relationships are derived by factoring the denominator of the fourth expression in equation s8, and then applying partial fraction expansion to equate the fourth and fifth expression in these equations.

$$\begin{matrix} (s9) A'_{f}=\frac{(A_{f}+A_{s}-B_{f}-B_{s})-\sqrt{(A_{f}+A_{s}-B_{f}-B_{s})^{2}-4(A_{f}A_{s}-A_{f}B_{s}-A_{s}B_{f})}}{2} \\ (s10) A'_{s}=\frac{(A_{f}+A_{s}-B_{f}-B_{s})+\sqrt{(A_{f}+A_{s}-B_{f}-B_{s})^{2}-4(A_{f}A_{s}-A_{f}B_{s}-A_{s}B_{f})}}{2} \\ \\ (s11) B'_{f}=\frac{A_{f}B_{s}+A_{s}B_{f}-(B_{f}+B_{s}+B'_{f})A'_{f}}{A'_{s}} \\ (s12) B'_{s}=B_{s}+B_{f}-B'_{f} \\ \\ (s13) x'_{f}(n+1)=A'_{f}x'_{f}(n)+B'_{f}f(n) \\ (s14) x'_{s}(n+1)=A'_{s}x'_{s}(n)+B'_{s}f(n) \\ (s15) x(n)=x'_{f}(n)+x'_{s}(n) \end{matrix}$$

For the error-dependent learning model presented in the paper, we estimated the learning rates and forgetting factors to be 0.2 and 0.59 for the fast system, and 0.02 and 0.99, for the slow system. The equivalent force-dependent learning system also has two learning states and similar fast and slow dynamics. Its fast learning component has a learning rate of 0.21 and a forgetting factor of 0.39, while its slow learning component has a learning rate of 0.01 and a forgetting factor of 0.98. Figure S2 shows the response of equivalent force-feedback and error-feedback learning systems. Note that the behavior of the net adaptation patterns of these two systems is equivalent, but the individual components within these responses are different. It is interesting to note that there is much better retention of the current state of learning when error is held to zero than if force were held to zero. This is what Sheidt et al (2001) found experimentally when they experimentally compared the retention of a learned force-field adaptation under these two conditions.

**Simulations of motor adaptation with the single-state, gain-specific, and multi-rate models on several motor adaptation paradigms.**

The results shown in figure s2 are a superset of those shown in figure 4 in the main text. **a-g**. anterograde interference. **h-n**. rapid unlearning. **o-u**. rapid downscaling. First column (**a,h,o**): experiment paradigms. Odd numbered rows (**b-d,i-k,p-r**): Raw simulation results. Blue: initial adaptation. Red, green and cyan: secondary adaptation after 30, 60, or 120 trials of the initial adaptation, respectively. Even numbered rows (**e-g,l-n,s-u**): Comparison of adaptation rates for initial and secondary adaptations. Here the learning curves have been shifted so that they all begin at zero and flipped so that the desired performance level is positive. In the anterograde interference paradigm (**a-g**), the multi-rate model correctly predicts that learning the opposite force-field proceeds with a slower time constant than initial learning, furthermore this time constant gets even slower when number of trials in the initial learning block is increased. However, the single-state model predicts that the initial and secondary adaptations proceed with the same rate while the gain-specific model predicts (incorrectly) that the rate secondary learning will be faster than that of initial learning. The multi-rate model also correctly predicts that unlearning proceeds with a faster time constant than initial learning (**k,n**) and the time constant for downscaling is faster still (**r,u**), however the time constant for unlearning or downscaling returns to baseline when the number of trials in the initial learning block is increased. In the unlearning and downscaling paradigms the single-state model incorrectly predicts a constant learning rate, while the gain-specific model correctly predicts a faster learning rate for the secondary adaptation. In summary, the multi-rate model simultaneously explains the effects of anterograde interference, rapid unlearning, and rapid down-scaling, while the single-state and gain-specific models cannot. Furthermore the multi-rate model makes testable predictions about these effects: anterograde interference will get stronger as the length of the initial adaptation period increases, but rapid unlearning and rapid-downscaling will get weaker as the length of the initial adaptation period increases.

**Figure S2.** Simulations of motor adaptation with the single-state, gain-specific, and multi-rate models on a variety of motor adaptation paradigms.

**Simulation of asymmetric gain-specific model of learning**

It is possible to extend Kojima’s gain-specific model so that each gain-specific learning process can have a different learning rate and retention factor. Although this asymmetric gain-specific model is somewhat different from how the gain-specific model was originally presented, it may be instructive to examine the behavior of this asymmetric gain-specific model, because it closely resembles our multi-rate model – both in the number of parameters and the meaning of those parameters (learning rates and retention factors for each of the two learning processes). Figure S3 shows that this asymmetric gain-specific model fails to produce the pattern of spontaneous rebound that we found in the error clamp experiment displayed in figure 3 in the main text. While the symmetric gain specific rebound fails to produce any rebound during the error clamp phase of this simulation, the asymmetric gain-specific model can predict some amount of rebound in error-clamp trials, but this rebound has a very different pattern to what we observed experimentally. Specifically, (1) this rebound is markedly greater in magnitude for one direction of initial learning than the other, and (2) the direction of rebound be will always be in the direction of the learning process with the higher retention factor (i.e. a particular bias direction), rather than consistently in the direction of the initial learning as predicted by the multi-rate model. However, our experimental results sharply disagree with this prediction, instead the experimental results clearly show that the direction of spontaneous rebound in the error clamp trials clearly changes when the direction of the initial learning is changed so that spontaneous rebound consistently occurs toward the direction of the initial learning rather than toward a particular bias direction (e.g. toward the right or toward the left). Furthermore, the magnitude of rebound is quite similar for both directions of initial learning (see figure 3). The multi-rate model, in contrast, agrees well with the data presented in figure 3. It consistently predicts spontaneous rebound in the direction of the initial learning, and it predicts that the magnitude of this rebound will be the same regardless of the direction of initial learning.

**Figure S3.** Simulation of spontaneous recovery for the multi-rate, symmetric gain-specific, and asymmetric gain-specific models in the error clamp experiment.

**Robustness of spontaneous rebound in the multi-rate model: simulation of the effects of different learning rates and forgetting factors on the amount spontaneous rebound predicted by the multi-rate model.**

A key feature of the multi-rate model is its ability to predict the spontaneous recovery that we found experimentally in the learning/unlearning/error-clamp paradigm (see figures 2 & 3). In the interest of conciseness, in the main paper we only showed spontaneous recovery for specific sets of model parameters, however spontaneous recovery is a general feature of this model over a very wide space of model parameters. Because an analytical approach to demonstrating this property is difficult, we performed a large set of simulations in which the model parameters where systematically varied (by as much as a factor of 10) from those that best fit our error clamp experiment data. In these simulations the fractional spontaneous recovery (max rebound/max initial learning) was assessed following asymptotic learning and unlearning to baseline. The results of these simulations are shown below. There are four parameters in the multi-rate model and each panel below displays the amount of spontaneous recovery when two of these parameters are systemically varied. There are six panels because there are six different two-parameter combinations. Note that in all cases more than 80% of the parameter space shown displays a spontaneous recovery of greater than 20%, where the amount of spontaneous recovery refers to the ratio of the maximum recovery in the error clamp phase to the asymptotic amount of learning during the initial learning phase. These simulations show that spontaneous recovery is a robust feature of the multi-rate model in this experimental paradigm, and that the finding of spontaneous recovery does not depend upon a narrow choice of parameter values. Note that $\bar{A}=1-A$ is shown on a log scale in the figure below rather than $A$, because learning and decay rates in the model vary directly with $\bar{A}$ rather than $A$.

**Figure S4.** Effect of parameter variation in the multi-rate model on the amount of spontaneous recovery predicted for the error-clamp paradigm.

**Fusi et al. (2005) model of synaptic plasticity and the phenomenon of savings**

Abbott and colleagues (Fusi et al. 2005) described a model of synaptic plasticity where each synapse may be in a strong or weak condition. Each condition was in turn supported by a set of states that had different levels of plasticity. When an event occurred that could potentially strength a synapse, the probability of state transition in the synapse depended on “depth” of its state. When the synapse was in a deep state, it was very unlikely that an event could change its state. Although Fusi et al. did not consider savings in the analysis of their model; the cascade nature of synaptic states encoded history of past events. We wondered whether such a structure could explain savings.

We considered a system of 200 synapses. As in the Fusi et al. model, each synapse could be either in a weak or a strong condition. When a synapse was in a weak condition it had a “value” of -1 and when it was in a strong condition it had a value of +1. Each synapse was supported by a cascade of states that could take a depth of 10, and the transition probability from one state to another was arranged in a geometric sequence, as described by Fusi et al.

We used an integer value that ranged from -10 to +10 to represent both the condition of a synapse (weak or strong) and its state (depth of 1 to 10),. For example, when the synapse was weak at state 3, it was labeled with -3. When the synapse was strong at state 5, it was labeled as +5. In this discussion, we will refer to the “state” of a synapse with this integer. However, the “value” of a synapse will remain either at -1 or +1 (that is, weak or strong). For example, when the synapses are scanned to read the content of the memory, we simply sum the values of all the synapses and divide the sum by the number of synapses to get the expected value of a synapse. This value will range from -1 to +1. On the other hand, the rate of change of this expected value will depend strongly on the distribution of the states of the synapses. The more the distribution is skewed to one extreme or the other, the more resistant it will be to change.

An important initial consideration is the steady state property of the system. As Fusi et al. had noted, if the probability of a synapse strengthening event is the same as a synapse weakening event, then the distribution of the synapse states will be uniform. Therefore, we began each simulation with the distribution of the synaptic states uniformly distributed.

In our first experiment with this system, we considered a scenario where the probability of a synaptic strengthening event was three times the probability of a synaptic weakening event. Figure S5A shows the expected value of the synapses for a typical run. After 200 events, the expected value has increased to around 0.75. At the beginning of the trials, the states of the synapses are uniformly distributed (Fig. S5B). At the end of the trials, the states cluster mostly on the high positive values, but some states remain highly negative (Fig. S5C).

In our second experiment, we asked whether the system showed interference. The system was driven by events that were three as probable to weaken the synapses vs. strengthen the synapses. As a result, the expected value of the synapses was initially driven negative (Fig. S6). After 300 events, the probability patterns were reversed to favor synaptic strengthening. We observed that after the weakening of the synapses, the learning rate in response to the strengthening events was reduced as compared to the naïve system. Therefore, the system exhibited history dependent learning rates. After a sequence of synaptic weakening events that drove the distribution of synaptic states to one extreme, the system was naturally much slower in responding to synaptic strengthening events.

Unfortunately for the Fusi et al. (2005) model, the history dependent learning rates produced the opposite behavioral results that one observes in savings. In our third experiment, we first trained the network with high probability of synaptic strengthening events (odds of 3 to 1), followed by a sharp reversal of the probabilities to drive the expected value of the system back to its initial condition (odds of 6 to 1 in favor of synaptic weakening event). We followed this with a test for savings in a sequence of strengthening events (odds of 3 to 1). We observed that the relearning was much slower than original learning (Fig. S7A). The reason for this is that the system is in its optimal state of learning when the distribution of states is uniform. After initial synaptic strengthening events, the synapses are mostly in a deeply positive state (Fig. S7B). When the events produce synaptic weakening with high probability, some but not all of the states shift toward negative values. As a result, by the end of the synaptic weakening phase, we have a bimodal distribution of states (Fig. S7C). At this point, the expected value of the system is zero, but the system has clearly been affected by the history of the past events. However, that history actually hinders performance when the system is provided with synaptic strengthening events: the only way to move the expected value of the system toward positive numbers is to change the negative states to positive. When the system is naïve, there are many negative states that are only slightly negative, and therefore they will move quickly. In our system, the training has left us with little or no shallow negative states. The negative states will take many strengthening events to move to positive values.

In summary, our simulations of the cascade model of synaptic memory suggest that while this system has strong history dependent plasticity properties, these properties do not include savings.

**Figure S5**. Adaptation to synaptic strengthening events. (A) Expected value of synapses during events that favor synaptic strengthening with odds of three to one. (B) Distribution of synaptic states at initial conditions. (C) Distribution of synaptic states at end of training.

**Figure S6.** Expected value of the synapses in a simulation where the system was initially trained with high synaptic weakening probability, followed by high synaptic strengthening probability. The rate of learning in the second phase is much slower than in the initial phase. The system shows interference.

**Figure S7**. An experiment on savings using the cascade model of synaptic plasticity. (A) Expected value of the synapses. The system was trained with high probability of strengthening events, followed by very high probability of weakening events, and then high probability of strengthening events. The rate of relearning was slower than initial rate of learning. (B) Distribution of synaptic states after initial strengthening events. (C) Distribution of synaptic states after the weakening events brought the expected value back to zero.
